# Galactokinase 1 Inhibition-Induced Cell Cycle Arrest and Apoptosis in Bladder Cancer Cells Is Associated with AKT Signaling Downregulation

**DOI:** 10.3390/ijms27062911

**Published:** 2026-03-23

**Authors:** Surya P. Singh, Ronghao Liu, Feng Yan, Qinggong Tang, Chinthalapally V. Rao, Venkateshwar Madka

**Affiliations:** 1 Center for Cancer Prevention and Drug Development, Stephenson Cancer Center, Hem-Onc Section, Department of Medicine, University of Oklahoma Health Campus, Oklahoma City, OK 73104, USA; surya-singh@ou.edu (S.P.S.); cv-rao@ou.edu (C.V.R.); 2 Stephenson School of Biomedical Engineering, University of Oklahoma, Norman, OK 73019, USA; ronghao.liu-1@ou.edu (R.L.); feng.yan@ou.edu (F.Y.); qtang@ou.edu (Q.T.); 3 VA Medical Center, Oklahoma City, OK 73104, USA

**Keywords:** galactokinase-1, cell cycle, apoptosis, bladder cancer, cpd36

## Abstract

Bladder cancer (BCa) is the second most common cancer of the genitourinary tract globally. It has limited treatment options, high recurrence rate, and acquires resistance to platinum-based therapy. Therefore, identifying novel therapeutic targets is urgently needed. Analysis of the TCGA data revealed that the enzyme galactokinase-1 (GALK1) is overexpressed (*p* < 0.0001) in bladder tumors compared to normal tissue. Our data also confirmed GALK1 protein upregulation in multiple human BCa cell lines and rodent bladder tumors. However, the precise role of GALK1 in BCa progression and effects of its specific inhibitor remain unexamined. In this study, we demonstrate that GALK1 gene silencing using shRNA resulted in a significant reduction in BCa cell proliferation, migration, and invasion. Pharmacological inhibition of GALK1 using small molecule Cpd36 resulted in anticancer efficacy against BCa. Cpd36 inhibited proliferation, migration, and invasion of BCa cells. Further, Cpd36 induced G1 phase cell cycle arrest, apoptosis, mitochondrial membrane depolarization, and ROS production in the BCa cells. Mechanistically, Cpd36-induced reduction in cell proliferation was associated with a decrease in expression of GALK1, PCNA proteins. Inhibition of metastatic potential was accompanied by decreased migration, invasion, and MMP-9 expression. Cell cycle arrest was associated with decrease in Cyclin D1 and increased expression of p21 and p27. Induction of apoptosis was linked with increased expression of cleaved caspase-3 and cleaved PARP, while downregulating p-AKT. Additionally, Cpd36 in combination with cisplatin or gemcitabine showed a strong synergistic effect on BCa cells. Taken together, our findings suggest that GALK1 plays a significant role in BCa cell survival and validates its inhibitors as promising therapeutic options for managing this disease.

## 1. Introduction

Bladder cancer (BCa) is still one of the significant global health concerns, ranking as the 10th most common cancer and the 6th most commonly diagnosed cancer among men [1]. According to estimates, 84,870 new cases of BCa will be diagnosed in the US in 2025, and 17,420 people are expected to die from the disease [2]. Smoking is a key risk factor for BCa, accounting for 50% of all cases in the U.S [3]. Currently, treatment options for BCa are limited to surgery, chemotherapy, and Bacillus Calmette-Guérin (BCG) therapy, which is an immunotherapy modality and has shown promising effects for early-stage BCa [4]. Chemotherapy with combination of gemcitabine and cisplatin is the standard treatment for BCa [5]. However, their effectiveness is limited by the development of resistance, and clinical trials with other antitumor drugs, including various molecularly targeted agents, have failed to demonstrate efficacy [6]. Despite this promising treatment, the majority of BCa patients die from distant metastases and tumor recurrences [7]. Therefore, the identification of novel therapeutic targets is urgently required.

New findings indicate that the altered metabolism of cancer cells may provide new targets for cancer interventions [8]. Metabolic reprogramming, particularly alterations in energy metabolism, is a hallmark of tumorigenesis [9]. Alterations in metabolic pathways within BCa cells provide crucial energy substrates and macronutrients for tumor growth [10]. In the Leloir pathway, galactose is phosphorylated by galactokinase 1 (GALK1) to produce galactose-1-phosphate. Moreover, galactose-1-phosphate undergoes an additional series of steps to be converted into glucose, a simple sugar that serves as the primary energy source for most cells [11]. Barretina and colleagues first documented that the GALK1 gene was significantly overexpressed in 28 different human liver cancer cell lines [12]. Upregulated GALK1 expression has been reported in patients with glioma compared to that in the normal human brain [13].

Recently, comparative proteomics studies have reported that the protein level of GALK1 in bladder cancer (BCa) tissues increases steadily with stage, indicating its potential as a biomarker for the early detection and prognosis of BCa [14]. These findings suggest that GALK1 is a crucial molecular target for BCa and plays a significant role in regulating metabolic homeostasis.

Also recently, a small-molecule inhibitor of GALK1, Cpd36, was developed that showed highly selective inhibition of galactokinase 1 [15]. Only a few studies have explored its potential anticancer activity. An earlier study showed that the GALK1 inhibitor Cpd36 decreased Glioblastoma Multiforme cell growth. Furthermore, this study also revealed that the presence of Cpd36 did not affect cells grown in glucose but significantly reduced the viability of cells grown on galactose [13]. However, no studies have explored the anticancer effects and associated mechanisms of Cpd36 in BCa.

In this study, we aimed to investigate possibility of metabolic modulation as therapeutic target in BCa and explore the underlying mechanisms by using the small-molecule GALK1 inhibitor Cpd36 against BCa. Furthermore, we examined the potential of combination therapy Cpd36 with cisplatin and gemcitabine in BCa cells. To our knowledge, this is the first study to assess the effects of Cpd36 in BCa cells toward validating Cpd36 and its target GALK1 pathway as a therapeutic agent for BCa.

## 2. Results

### 2.1. GALK1 Is Overexpressed in BCa Cells and Tumor Tissues

**Figure 1:**
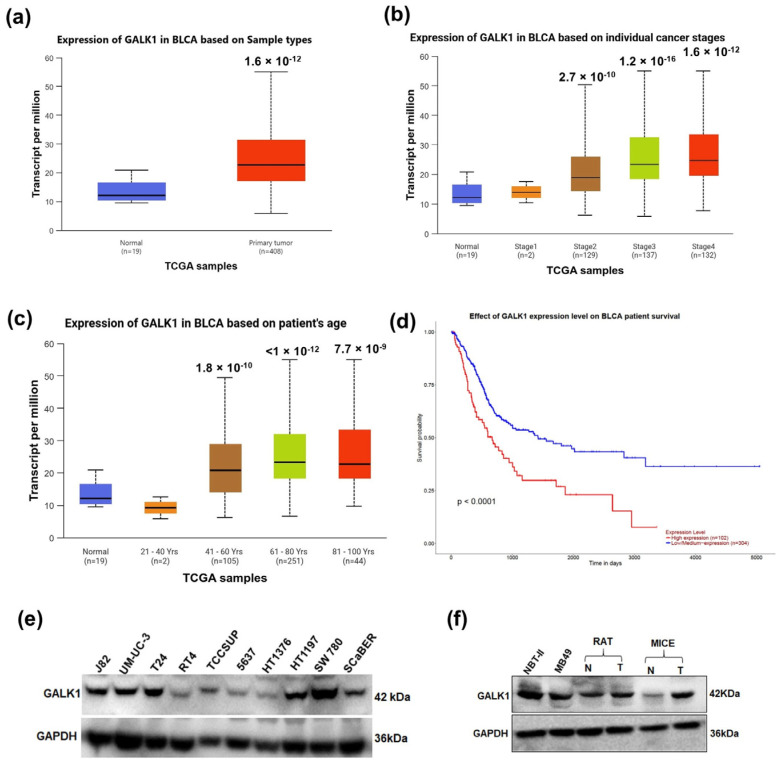
GALK1 overexpression is associated with poor prognosis in BCa. (**a**) TCGA dataset analysis showing the expression of GALK1 in normal and bladder tumor tissues. (**b**,**c**) Expression of GALK1 at various stages of BCa and across different age groups of patients. (**d**) The expression of GALK1 and survival in bladder cancer patients. (**e**,**f**) Western blot showing GALK1 protein expression in multiple BCa cell lines and (**f**) rodent tumors.

To examine expression level of GALK1 and its correlation with disease stage and prognosis in patients with BCa, we analyzed TCGA data using the UALCAN web portal. The results revealed that GALK1 expression was significantly elevated in BCa tumor samples compared to that in normal tissues (*p* < 1.65 × 10^−12^; Figure 1a). Furthermore, we observed that significant overexpression of GALK1 was associated with advanced stages of bladder cancer and patient age (Figure 1b,c). The TCGA data also revealed a correlation between GALK1 expression and patient survival; higher levels of GALK1 gene expression were associated with a poorer survival rate (*p* < 0.0001) compared with lower GALK1 expression (Figure 1d). Next, we performed immunoblot analysis to examine GALK1 expression in BCa cell lines and bladder tumors. Elevated GALK1 expression was observed in multiple BCa cells (Figure 1e) and bladder tumor tissues from rodent models compared to corresponding normal bladder tissues (Figure 1f). Collectively, these findings indicate that BCa cells may have evolved metabolic adaptation to utilize an alternate energy source by overexpressing GALK1, which may further contribute to the tumor promotion and poor prognosis of BCa patients.

### 2.2. shRNA-Mediated GALK1 Knockdown Inhibits BCa Cell Survival

**Figure 2:**
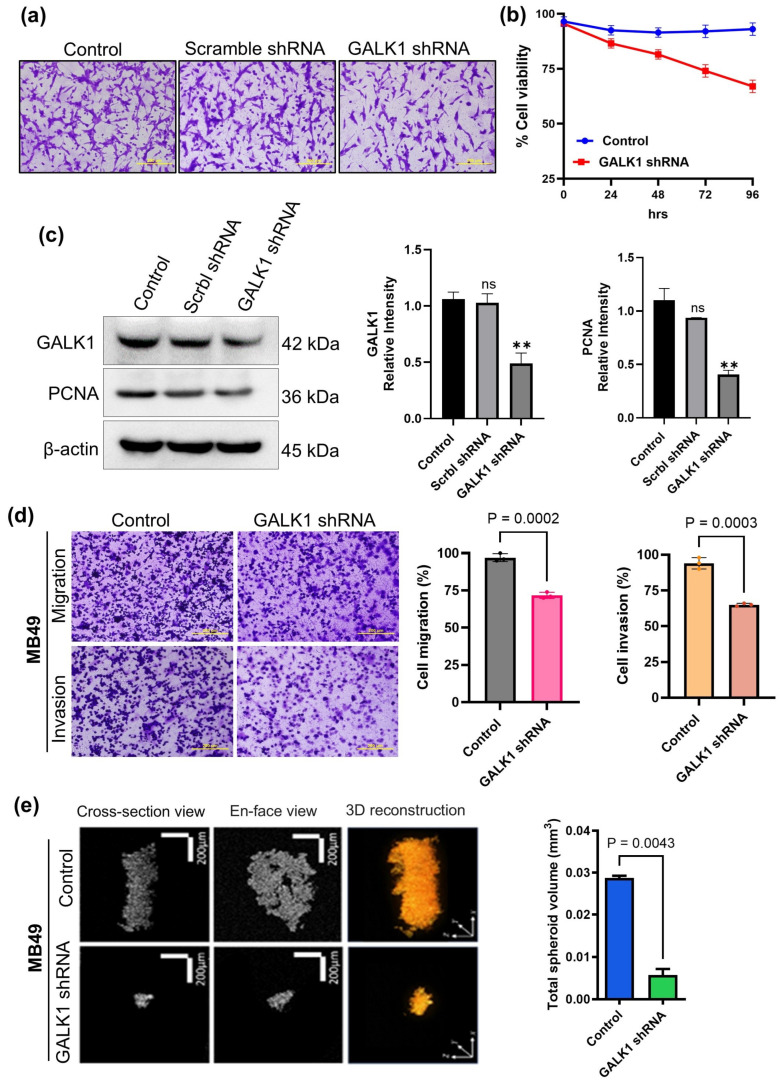
GALK1 knockdown decreased cell proliferation and inhibited the migration and invasion of BCa cells. (**a**) Microscopic image of the MB49 cells with knockdown of GALK1 expression using shRNA. (**b**) Trypan blue assay showing cell viability after GALK1 knockdown at different time points in MB49 cells. (**c**) Western blot analysis was performed to assess the levels of GALK1, PCNA, and β-actin in GALK1 knockdown MB49 cells. (**d**) Transwell migration and invasion assays were conducted to evaluate the migratory and invasive potential of BCa cells after GALK1 knockdown. (**e**) Representative OCT images and 3D volume renderings of MB49 cells-derived spheroids after 3 days of growth. Data shown are mean ± SEM of triplicate (*n* = 3) samples for each treatment. ** *p* < 0.01, and ‘ns’ for no significance.

To investigate the biological role of GALK1 in BCa cells, we used shRNAs to knockdown GALK1 expression in MB49 mouse BCa cells. Microscopy and viability data suggested reduction in cancer cell proliferation in shRNA transfected cells (Figure 2a,b). Protein expression analysis confirmed decreased expression of GALK1 as well as cell proliferation biomarker PCNA in shRNA transfected MB49 cells (Figure 2c). Next, we assessed the migration and invasion potential of GALK1 knockout MB49 cells using Transwell migration and invasion assays. As demonstrated in Figure 2d, the knockdown of GALK1 in MB49 cells significantly decreased the migration and invasion abilities of the cells. Next, we grow BCa spheroids from MB49 and GALK1 knockdown MB49 cells to assess the growth in the 3D model of BCa. OCT imaging analysis revealed a reduction in spheroid size and structural integrity in GALK1 knockdown MB49 spheroids, as demonstrated by disrupted internal morphology and fragmented 3D architecture (Figure 2e). This data indicated that GALK1 may contribute to cancer cell survival, and targeting this enzyme can have suppressive effect on proliferative and invasive properties of BCa cells.

### 2.3. GALK1 Inhibition Abrogates BCa Cell Growth, Migration, and Invasion

**Figure 3:**
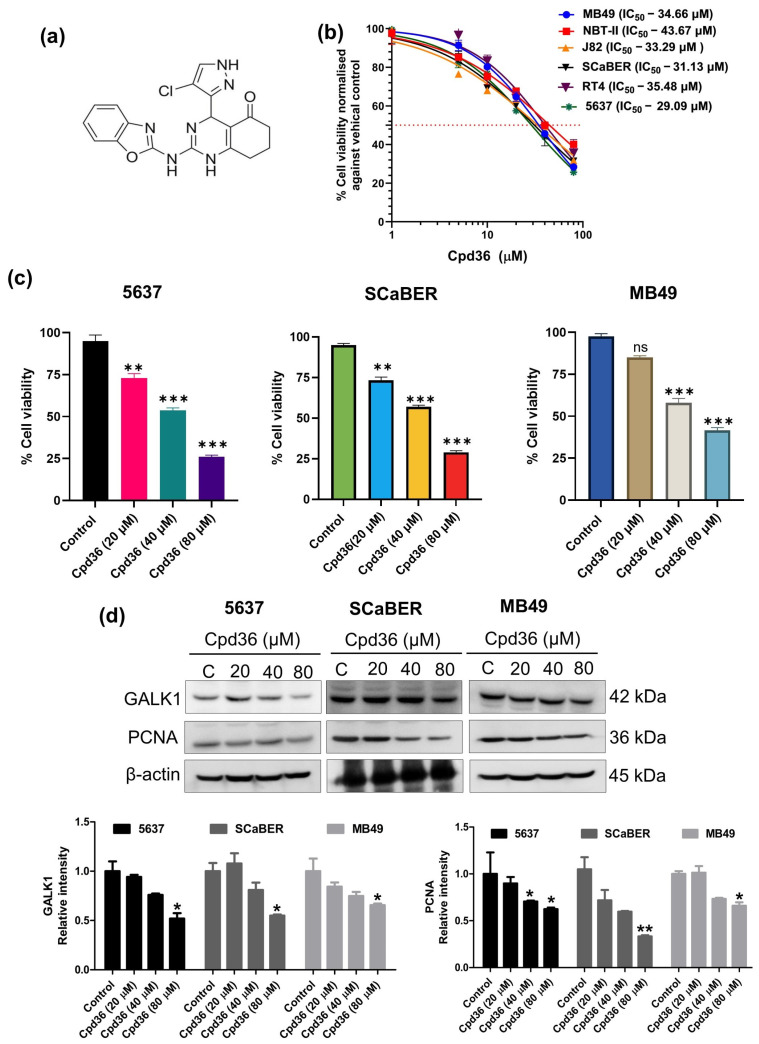

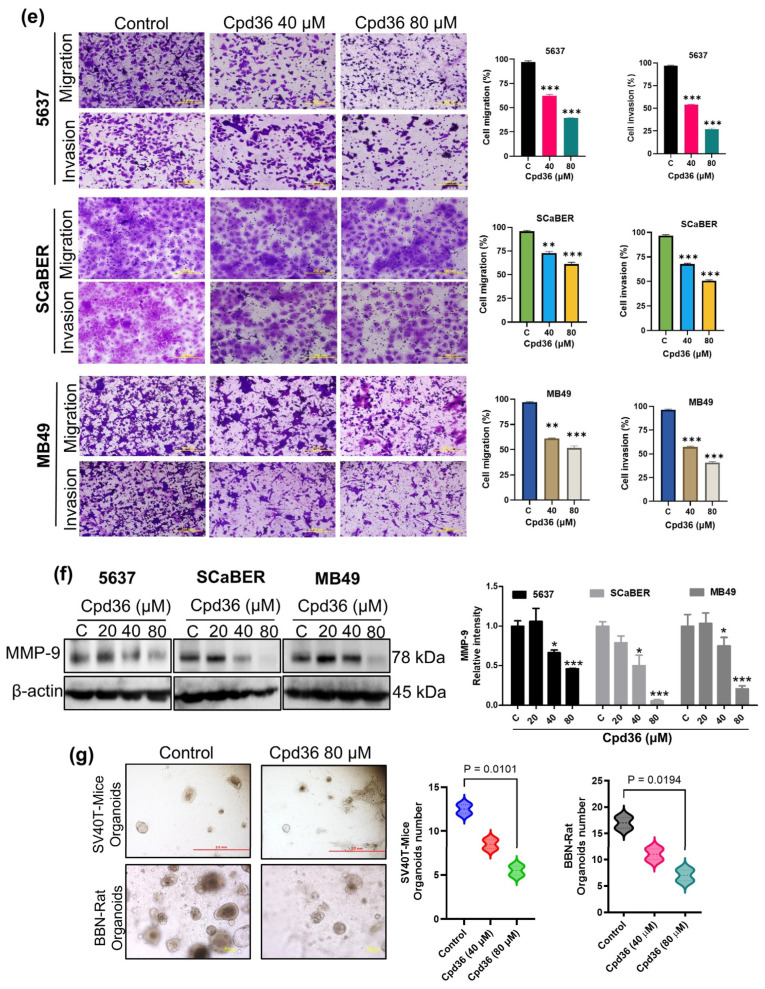
GALK1 inhibitor Cpd36 inhibits the proliferation, migration, and invasion of BCa cells. (**a**) Chemical structure of GALK1 inhibitor Cpd36. (**b**) MTT assay was used to assess the growth-inhibitory effect of Cpd36 on BCa cells after 48 h of treatment. Red dotted line represents IC50. (**c**) 5637, SCaBER, and MB49 cells were treated with 20–80 µg∕ml for 48 h, and the percentage of cell viability was calculated using trypan blue dye. (**d**) Western blot analysis was performed to examine the protein expression of GALK1 and PCNA in BCa cells. (**e**) Representative image showing the Transwell migration and invasion assay of BCa cells after 48 h of Cpd36 treatment. (**f**) Western blot analysis of MMP-9 and β-actin in BCa cells. (**g**) Effect of Cpd36 on UPII-SV40T tumor-derived organoids and BBN-rat tumor-derived BCa organoids, magnification 20×; Scale bar, 200 µm. The results are representative of three replicates (*n* = 3) mean ± SEM. Significance is indicated by * *p* < 0.05, ** *p* < 0.01, *** *p* < 0.001, and ‘ns’ for no significance.

As shown above, GALK1 is overexpressed in BCa, and its knockdown demonstrated antiproliferative effects in cancer cells. We aimed to determine whether the small-molecule inhibitor of GALK1 Cpd36 (Figure 3a) could suppress BCa cell growth. To investigate this, six different BCa cell lines were treated with Cpd36, and cell viability was assayed. Data indicated that Cpd36 treatment significantly reduced cell viability, with an IC50 range of ~36 ± 7 µM for all cell lines tested (Figure 3b). To further explore the anticancer-associated molecular mechanism of Cpd36, two human cell lines (5637, SCaBER) and one murine (MB49) BCa cell line were selected and treated with Cpd36 (0–80 μM) for 48 h. Trypan blue staining showed that Cpd36 treatment increased cell death in 5637, SCaBER, and MB49 cells in a dose dependent manner (Figure 3c). Western blot analysis showed that treatment with Cpd36 significantly decreased the protein expression of GALK1 and PCNA, further supporting the antiproliferative effects of Cpd36 on BCa cells. (Figure 3d).

Additionally, Transwell migration and invasion studies indicated that Cpd36 treatment inhibits the migration and invasion capabilities of BCa cells (Figure 3e). Furthermore, Cpd36 treatment reduces MMP-9 expression in BCa cells, demonstrating its inhibitory effect on metastatic potential (Figure 3f). We also assessed the impact of Cpd36 on the growth of organoids derived from mouse and rat bladder tumor tissues. Results indicate that Cpd36 treatment decreased the number of mice and rat-derived organoids after 6 days compared to the control (Figure 3g). These findings suggest that inhibiting GALK1 with the small-molecule inhibitor Cpd36 may offer a promising treatment strategy for inhibiting BCa cell proliferation and metastasis.

### 2.4. Cpd36 Induces G1 Phase Cell Cycle Arrest and Apoptosis

**Figure 4:**
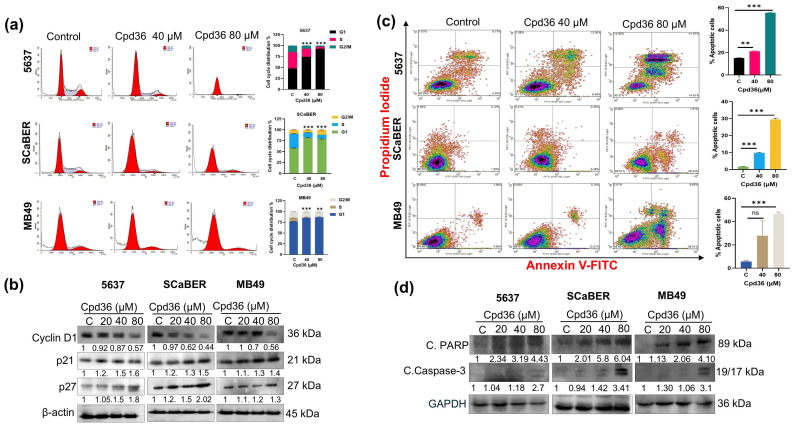
Cpd36 arrests the cell cycle and induces apoptosis in BCa cells. (**a**) Flow cytometry analysis showing cell cycle progression in 5637, SCaBER, and MB49 cells treated with Cpd36 for 48 h. The cell populations in the G1, S, and G2/M phases were determined. (**b**) Western blot analysis was performed to assess Cyclin D1, p21, and p27 expression, with the membranes reprobed for β-actin to verify equal protein loading. (**c**) Annexin V staining was performed to detect the induction of apoptosis in BCa cells. (**d**) Western blot analysis was performed to detect the apoptosis markers cleaved-PARP and cleaved-caspase-3 in BCa cells. The densitometric value for each protein is quantified using ImageJ software 1.54r and is displayed below each band as the fold change relative to the control. ** *p* < 0.01, *** *p* < 0.001, and ‘ns’ for no significance.

To test whether the Cpd36 growth-inhibitory effect is associated with cell cycle arrest, we performed cell cycle analysis using flow cytometry. As illustrated in Figure 4a, treatment with Cpd36 (40 and 80 μM) at 48 h resulted in a concentration-dependent increase in the number of cells in the G1 phase, along with a significant decrease in the S phase in 5637, SCaBER, and MB49 cell lines. We further validated the cell cycle-inhibitory effect of Cpd36 by performing Western blot analysis of cell cycle regulatory proteins, including cyclin D1, p21/Cip1, and p27/Kip1. Our results showed that Cpd36 treatment reduced cyclin D1 levels while increasing p21/Cip1 and p27/Kip1 expression compared with the control group (Figure 4b). These findings suggest that the modulation of these cell cycle regulators could mediate Cpd36-induced G1 arrest in BCa cells.

Next, we examined the effect of Cpd36 on apoptotic pathways in BCa cells. We used a dual staining method, Annexin V-FITC/propidium iodide (PI), to evaluate apoptosis induction in the 5637, SCaBER, and MB49 cell lines. The results indicated that treatment with Cpd36 (40 and 80 μM) for 48 h increased the percentage of apoptotic cells in a dose-dependent manner in 5637, SCaBER, and MB49 cells (Figure 4c). To further support this data, we examined the expression of apoptotic marker proteins via Western blot analysis. A considerable increase in cleaved caspase 3 and cleaved PARP expression was observed in 5637, SCaBER, and MB49 cells after treatment with Cpd36 (Figure 4d). Collectively, these data suggest that Cpd36 causes G1 phase cell cycle arrest and induces apoptosis in BCa cells.

### 2.5. Cpd36 Induces Mitochondrial Dysfunction

**Figure 5:**
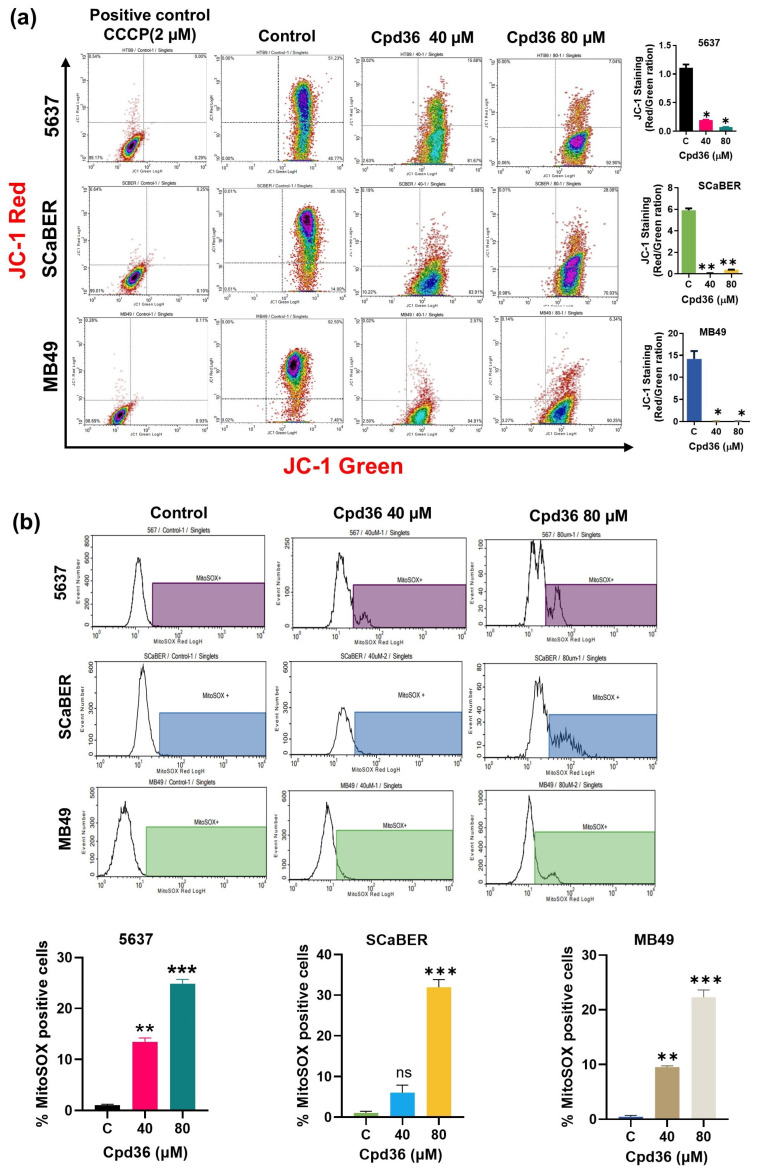
Cpd36 decreased the mitochondrial membrane potential and induced mitochondrial ROS production in BCa cells. (**a**) JC-1 staining was used to assess the mitochondrial membrane potential in 5637, SCaBER, and MB49 cells treated with Cpd36 or CCCP (as a positive control) for 48 h. (**b**) The fluorescence intensity of mitochondrial O2– was measured using MitoSOX^TM^ Red after 48 h of Cpd36 treatment. Data are presented as mean ± SEM, with significance levels indicated as * *p* < 0.05, ** *p* < 0.01, *** *p* < 0.001, and ns for no significance.

To assess the impact of Cpd36 on mitochondrial membrane potential (mtMP) 5637, SCaBER, and MB49 cells were stained with JC-1, a fluorescent probe, and analyzed by flow cytometry. Our observations showed that the shift from red to green fluorescence reflected a decrease in mtMP in BCa cells treated with the cytotoxic dose of Cpd36 (40 and 80 μM) or the positive control CCCP (2µM) (Figure 5a). Mitosox Red fluorescent dye was used to measure mtROS to determine whether the changes in mtMP were induced by mtROS production. The analysis indicated that cells treated with cytotoxic dose Cpd36 (40, 80 μM) showed a dose-dependent increase in ROS production in the 5637, SCaBER, and MB49 cell lines (Figure 5b). These data indicate that Cpd36 induces apoptosis in BCa cells via mitochondrial membrane potential depolarization and ROS production, which may be one mechanism contributing to cell death in BCa.

### 2.6. Cpd36 Modulates Akt Signaling

**Figure 6:**
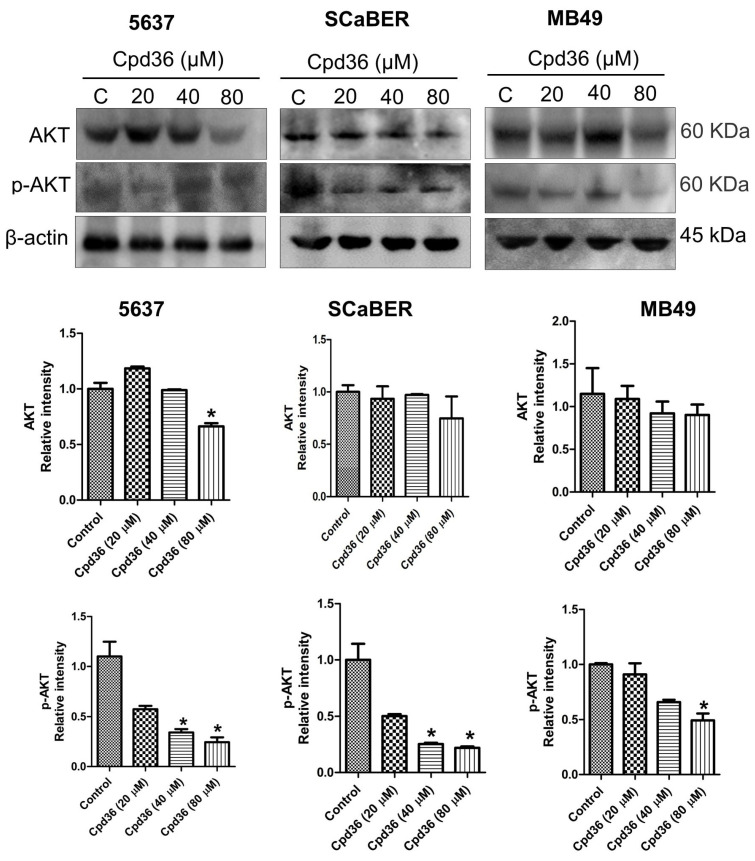
Cpd36 effects Akt signaling in BCa cells. Western blot analysis of p-AKT, AKT, and β-actin protein levels in 5637, SCaBER, and MB49 cells after treatment with Cpd36 for 48 h using appropriate primary and secondary antibodies. Western blot data are shown as mean ± SEM from two replicates (*n* = 2). * *p* < 0.05.

To further investigate the anticancer mechanism of Cpd36 in BCa, we analyzed the AKT and phosphorylated AKT (pAKT) levels using Western blot. Treatment of cells with Cpd36 resulted in a significant reduction in AKT phosphorylation. However, the total expression of AKT decreased in higher dose (80µM) of Cpd36 in all these cell lines, 5637, SCaBER, and MB49 cells (Figure 6). Thus, our findings suggest that the inhibition of the AKT signaling pathway could be involved in the reduction in BCa growth.

### 2.7. Combination of Cpd36 with Chemotherapy Drugs Synergistically Inhibits BCa Growth

**Figure 7:**
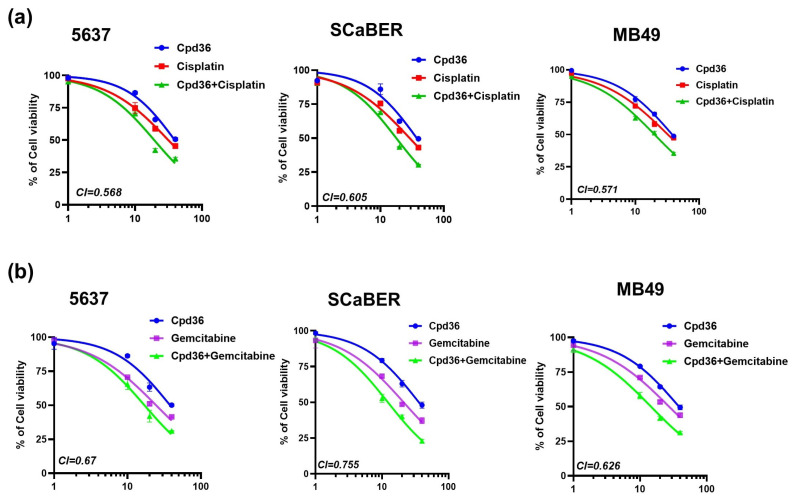
Cpd36 treatment sensitized BCa cells to chemotherapy drugs. 5637, SCaBER, and MB49 cells were incubated with Cpd36, or its combination with cisplatin (**a**) or gemcitabine (**b**), for 48 h. Cell viability was determined using the MTT assay. Combination index values were calculated and are mentioned in the graph for the combination of Cpd36 and cisplatin or gemcitabine. Data is shown as the mean± SEM from three independent experiments.

To examine the potential effects of Cpd36 in combination with the chemotherapy drugs cisplatin and gemcitabine on BCa cells. First, we treated 5637, SCaBER, and MB49 cells with Cpd36, cisplatin, or a combination of both for 48 h. Cell viability was evaluated using the MTT assay. The results showed that the combination of Cpd36 with cisplatin treatment reduced cell proliferation compared to individual treatments (Figure 7a). Similarly, cells treated with a combination of Cpd36 and gemcitabine for 48 h showed a more synergistic cytotoxic effect on 5637, SCaBER, and MB49 cells (Figure 7b). The combination index (CI) was calculated using the Chou-Talalay method. We observed a CI value of less than 1, indicating a synergistic effect between Cpd36 and the growth-inhibitory effects of gemcitabine and cisplatin. These results suggest that the combination of Cpd36 and gemcitabine has a more significant synergistic cytotoxic effect on BCa cells than the combination of cisplatin.

## 3. Discussion

Several alterations in metabolic pathways are associated with the pathogenicity of BCa, implicating modulation of metabolic pathways may offer therapeutic or preventive benefits. Identifying the metabolic alterations caused by a particular oncogene is essential for developing a treatment that selectively targets the tumor metabolism. Studies have reported that GALK1 is overexpressed in various types of cancer [12,13]. A recent study revealed that GALK1 serves as a biomarker for the early detection and prognosis of BCa [14]. Our findings also support that GALK1 expression is higher in BCa tissue and various BCa cell lines, suggesting the oncogenic role of GALK1 in bladder cancer. Tang et al. reported that knockdown of the GALK1 gene by siRNA suppressed the growth of HepG2 cells [16]. Similarly, our data showed that silencing the GALK1 gene with shRNA inhibited the growth of MB49 cells. Only a few inhibitors, such as NCATS-SM4487 and Cpd36, have been developed as highly selective inhibitors of GALK1 [15]. However, no studies in the existing literature have reported the use of GALK1 inhibitor Cpd36 against BCa. In the present study, we hypothesized that inhibition of the Leloir pathway enzyme GALK1 with Cpd36 could be a promising therapeutic approach for the treatment of BCa. Cpd36 is a selective inhibitor of GALK1 discovered through high-throughput screening and has been shown to inhibit the growth of GBM cells [13]. Metastatic skin cutaneous melanoma and breast cancer have been found to use the galactose metabolic pathway [17]. In vivo findings showed that aggressive breast cancer cells with galactose upregulate the enzymes of the Leloir pathway [17,18]. Our findings indicate that Cpd36 also inhibits the growth of 5637, SCaBER, and MB49 cells by reducing the expression of GALK1 and PCNA, suggesting the inhibition of the Leloir pathway enzyme in BCa.

Uncontrolled cell growth is a central characteristic of cancer, and PCNA is a crucial regulator of this process [19]. The migration and invasion of cancer cells are crucial for metastatic disease, which accounts for the majority of cancer-related deaths [20]. We found that Cpd36 treatment inhibits the migration and invasion of 5637, SCaBER, and MB49 cells. MMP-9 is a type IV collagenase that belongs to the gelatinase group of human matrix metalloproteinases (MMPs). It plays a crucial role in degrading the extracellular matrix (ECM), facilitating the migration and invasion of transitional cancer cells to distant sites [21]. Research has also shown that MMP-9 is overexpressed in BCa and is associated with a higher tumor grade [22]. Our findings showed that Cpd36 significantly reduced MMP-9 expression, highlighting the antimetastatic effect of Cpd36 in BCa cells. Thus, this finding provides evidence that Cpd36′s inhibitory effect on migration and invasion in BCa cells is associated with its inhibition of MMP-9. Several studies have shown that 3D models are valuable tools for examining drug penetration and efficacy and better mimic in vivo environmental conditions than two-dimensional cancer models [23,24,25]. Our findings showed that a higher dose of Cpd36 decreased BCa spheroids and organoids, suggesting a strong anti-proliferative effect on BCa.

Cell cycle dysregulation and resistance to apoptosis are both characteristics of cancer [26]. The development of cancer involves the accumulation of mutations or alterations in the genes that regulate the cell cycle, thereby disrupting the normal regulation of cell growth and division [27]. The present study showed that Cpd36 induced G1 phase cell cycle arrest in 5637, SCaBER, and MB49 BCa cells, indicating that Cpd36 impairs cell cycle progression in BCa. Cell cycle regulatory molecules enhance the transition from the G1 to S phase in cancer cells, promoting cell proliferation. Cyclin D1 is an important cell cycle regulator during the G1 phase, and its overexpression or dysregulation is strongly associated with cancer pathogenesis [28]. Since Cpd36 inhibits G1 phase cell cycle progression in BCa, we analyzed the expression of cyclin D1 in 5637, SCaBER, and MB49 BCa cells. Western blotting data showed that cyclin D1 was downregulated after Cpd36 treatment, further supporting G1 phase cell cycle arrest. The Cip/Kip family members of CDK inhibitors, p21 and p27, function as tumor suppressors by regulating the progression of cells from the G1 phase to the S phase [29]. Several studies have reported that these two CDK inhibitors are upregulated during cell cycle arrest in response to various anticancer agents [30,31]. Our results suggest that Cpd36 treatment mediates an important anticancer mechanism, including decreased cyclin D1 and increased p21/cip1 and p27/kip1 levels in BCa cells.

Evidence indicates that apoptosis often occurs in cells during the G1 phase of the cell cycle, and that an arrest in late G1 or S phase can accelerate apoptosis [32]. Apoptosis is a complex and regulated process of programmed cell death that is essential for maintaining tissue homeostasis and supporting proper developmental processes. Caspase 3 is a widely studied protease that plays a central role in executing apoptosis, responsible for cleaving PARP in cells undergoing apoptosis [33]. Cpd36-induced cell death in 5637, SCaBER, and MB49 cells was associated with the cleavage of caspase 3 and PARP, highlighting that Cpd36 has a significant role in apoptosis induction.

We analyzed the mechanism of Cpd36-induced apoptosis. Accumulating evidence suggests that a decrease in mitochondrial membrane potential can lead to the production of reactive oxygen species (ROS), resulting in substantial oxidative damage [34]. The damage involves lipids, nucleic acids, and proteins, resulting in irreversible apoptosis. Cpd36 significantly elevated intracellular ROS levels and induced a loss of mtMP in BCa cells. This finding indicates that mitochondrial dysfunction may occur during apoptosis induced by Cpd36. This highlights the essential role of mitochondria in the induction of apoptosis. However, further mechanistic studies are needed to elucidate the mechanisms underlying mitochondrial dysfunction associated with GALK1 inhibition.

Akt serine/threonine kinase, also known as protein Kinase B (PKB), is the most activated oncogenic protein in cancer [35]. Akt is activated by phosphorylation at an essential site on Thr308 or Ser473, and phosphorylates several downstream protein substrates, such as mouse double minute 2 homolog, Bcl-2-associated death promoter, forkhead in rhabdomyosarcoma, and GSK3b [35,36]. Phosphorylated AKT (pAKT) is involved in the deregulation of cell proliferation, cell cycle progression, motility, apoptosis, and metabolism in cancer cells [36]. AKT signaling regulates inflammation and tumor formation in BCa and promotes resistance to chemotherapy drugs, such as paclitaxel [37]. Considering the role of AKT in BCa, we investigated the effect of Cpd36 on AKT signaling. Our findings showed that Cpd36 treatment significantly suppressed the phosphorylation of Akt in BCa cells. Thus, suggesting that the inhibition of AKT signaling may be involved in the suppression of BCa growth.

Understanding the metabolic reprogramming associated with drug resistance is essential because resistance to chemotherapy or targeted molecular therapy is a significant cause of tumor recurrence and death [38]. Cisplatin-based chemotherapy is the standard treatment for metastatic BCa; however, its clinical use is limited because of the high rate of chemoresistance [39]. Gemcitabine (GEM) is another antimetabolite drug used for the treatment of metastatic BCa. However, GEM resistance presented a significant challenge in the treatment of muscle-invasive bladder cancer (MIBC). The response rate of advanced MIBC with GEM treatment is less than 40%, suggesting the limited efficacy of GEM treatment [40,41]. Our findings demonstrate that GALK1 inhibition by Cpd36 enhances the sensitivity of BCa cells to both chemotherapy drugs, cisplatin and gemcitabine, suggesting that it may be beneficial in addressing current clinical challenges associated with reversing drug resistance in BCa. This hypothesis requires additional mechanistic studies to gain a deeper understanding of its role and potential implications in BCa.

## 4. Materials and Methods

### 4.1. Bioinformatics Tools

We utilized UALCAN (https://ualcan.path.uab.edu) as an interactive web resource for analyzing GALK1 expression in bladder cancer [42]. The expression of the GALK1 gene was examined and visualized in normal tissues (N = 19) and bladder tumor tissues (N = 408) using box plots. The clinical information, including bladder cancer stage, patient age, and survival, was also analyzed using this web resource.

### 4.2. Cell Culture

All BCa cell lines were purchased from the American Type Culture Collection (ATCC, Manassas, VA, USA), except for MB49. MB49 mouse BCa cells were obtained from Accegen, Fairfield, NJ, USA (ABC-TC2235S) and cultured in DMEM (ATCC # 30-2002) media. Human BCa cells J82 (ATCC # HTB-1), UMUC-3 (ATCC # CRL-1749), TCCSUP (ATCC # HTB5), HT1376 (ATCC # CRL-1472), HT1197 (ATCC # CRL-1473), 5637(ATCC # HTB9), SCaBER (ATCC # HTB3) and rat BCa cells NBT-II (ATCC # CRL-1655) were grown in Eagle’s Minimum Essential Medium (EMEM) (ATCC # 30-2003). McCoy’s 5A Medium Modified (ATCC # 30-2007) were used to culture the T24 (ATCC # HTB4), RT4 (ATCC # HTB2) cells. 5637(ATCC # HTB9) grown in RPMI-1640 (ATCC # 30-2001) while SW780 (ATCC # CRL-2169) was cultured in Leibovitz’s L-15 (ATCC # 30-2008) media. All cell culture media were supplemented with 10% fetal bovine serum and 1% penicillin and streptomycin. Cells were grown in a CO_2_ incubator.

### 4.3. shRNA Transfection in MB49 Cells

MB49 cells were seeded at 2 × 10^5^ cells per well in a 6-well tissue culture plate and incubated in a CO_2_ incubator until the cells reached 60–80% confluence. shRNA (SC Cat # 60672-SH) and transfection reagent solutions were prepared according to the manufacturer’s protocol (Santa Cruz Biotechnology, Dallas, TX, USA). Cells were then washed with 2 mL of shRNA transfection medium (SC Cat # 108062), and the prepared solution containing 200 µL shRNA plasmid DNA/shRNA transfection reagent complex was added to the cells and incubated in a CO_2_ incubator for 5–7 h. At the end of the incubation period, 1 mL of DMEM media containing 2X FBS and antibiotic concentrations was added, and the cells were incubated in CO_2_ for an additional 18–24 h. The medium was aspirated, and normal DMEM was added. The cells were then processed for Western blot analysis.

### 4.4. MTT Assay

BCa cell lines were cultured in a 96-well plate at a density of 8 × 10^3^ cells per well, and Cpd36 (Sigma, St. Louis, MO, USA Cat # 5339190001) drug was prepared in 0.1% DMSO and treated at a concentration range of (0–80 μM) for 48 h. For the combination study, cells were treated with different concentrations (0–80 μM) of Cpd36, cisplatin, or gemcitabine, either individually or in combination, under the same incubation conditions for 48 h. After the incubation period, MTT solution was added to each well, and the plates were placed in the CO_2_ incubator for an additional 3 to 4 h. After discarding the MTT solution, 200 µL of DMSO was added to each well to dissolve the formazan crystals. The absorbance was then measured at 570 nm with an ELISA reader (Scientific Multiskan MK3, Thermo Fisher Scientific, Waltham, MA, USA). The IC50 value was calculated from dose–response curves using nonlinear regression analysis (log [inhibitor] vs. response–variable slope) with GraphPad Prism software. Each value represents the mean ± SEM from three independent replicates (*n* = 3).

### 4.5. Spheroid Culture

MB49 and GALK1 knockout MB49 cells were grown in DMEM medium under a CO_2_ incubator. When cells reached 70% confluence, they were trypsinized, and 2 × 10^3^ cells/well were seeded in a 96-well ultra-low attached plate in 200 µL media and incubated in a CO_2_ incubator for 3 days. After completion of the incubation time, BCa spheroids and fixed with 4% formalin and subjected to OCT imaging.

### 4.6. OCT Imaging of Bladder Cancer Spheroids

Optical coherence tomography (OCT) imaging was performed using a polarization-sensitive spectral-domain OCT system (Telesto, Thorlabs, Newton, NJ, USA center wavelength, 1300 nm; bandwidth, 100 nm) [43]. The system provides an axial resolution of 5.5 µm in air (≈4.1 µm in tissue, *n* = 1.35) and a lateral resolution of 11 µm, with a sensitivity of ~100 dB and a maximum imaging depth of 3.5 mm. Volumetric datasets were acquired using a raster scanning protocol (200 × 200 A-scan grid) at an A-scan rate of 76 kHz. The samples were immersed in PBS during imaging to minimize the refractive index mismatch and maintain spheroid hydration. Raw B-scans were precisely pre-processed to enhance the speckle contrast and optimize the spheroid segmentation. A Pix2Pix-based deep-learning model was trained to produce foreground probability maps using manually annotated OCT slices. The resulting masks were refined through standard morphological operations to remove isolated noise and enforce structural continuity. Importantly, these uniform preprocessing steps preserved the integrity of the OCT intensity values during volumetric analysis. For visualization, 3D reconstructions were generated in Amira 2019.2 using fixed opacity and lighting profiles to ensure consistent appearance across samples. Renderings were used solely for qualitative assessment and not for quantitative analysis. No geometric smoothing or resampling was applied to avoid introducing artificial structural features.

Spheroid volumes were quantified directly from 3D segmentation masks derived from preprocessed OCT datasets. For each volumetric stack, a binary foreground mask delineating the spheroid boundary was generated using the Pix2Pix-based segmentation pipeline described above and subsequently refined with morphological operations to enforce boundary continuity. Only the largest 3D connected component was retained to eliminate isolated noise fragments and ensure anatomical validity. The physical voxel dimensions were determined from the OCT system calibration and refractive-index correction (5 µm × 5 µm × 2.54 µm for the x, y, and z axes, respectively; refractive index *n* = 1.35). Total spheroid volume V was computed by counting the number of voxels classified as foreground (*N*vox) and multiplying by the physical voxel volume:

V= *N*vox × (dx × dy × dz).

No geometric smoothing, interpolation, or resampling was performed prior to volume computation to avoid altering spheroid morphology. All samples were processed using identical segmentation and quantification parameters to ensure consistency and comparability across experimental groups.

### 4.7. Tumoroids Culture

Carcinogen BBN-induced BCa rat and UPII-SV40T transgenic bladder cancer mouse models were used to collect bladder tumor tissue. Bladder tumors collected from both models were cut into small pieces using a sterile surgical blade and washed with PBS. Tumor tissues were incubated with a solution containing 1 mg/mL collagenase and a ROCK inhibitor (MedChemExpress, Monmouth Junction, NJ, USA # Y-27632) in Dulbecco’s modified DMEM/F-12 medium and incubated at 37 °C for 30 min. After incubation, the cell suspension was filtered through a sterile 70-micron filter in a 50 mL falcon tube and centrifuged at 1800 rpm for 5 min. The supernatant was discarded, and the pellet was dissolved in fresh DMEM/F-12 medium. Cells were counted using Trypan blue. Live cells (1 × 10^5^) were mixed with 1 mL of basement membrane extract (BME) at a 1:1 ratio, plated in a 24-well plate, and incubated for an additional 20–30 min. After the BME solidified, complete DMEM/F-12 medium containing 100 ng/mL of FGF10 (Peprotech, Rocky Hill, CA, USA # 100-26), 25 ng/mL of FGF7 (Peprotech, Rocky Hill, CA, USA # 100-19), A83-01 (MedChemExpress # HY-10432), 500 nM of ROCK inhibitor (MedChemExpress, Monmouth Junction, NJ, USA # Y-27632), and B27 (Gibco, Carlsbad, CA, USA # 17504044) was added and incubated in CO_2_ incubators. After 24 h, Cpd36 drugs were added at concentrations of 40 μM and 80 μM for 6 days, organoid growth was monitored, and images were captured using bright-field microscopy.

### 4.8. Cell Migration and Invasion Assay

Cell migration and invasion were measured using Transwell chambers and Matrigel-coated polycarbonate filters, respectively. For migration assay, 1 × 10^5^ cells were plated in the upper chamber of an 8-micron Transwell in serum-free media containing Cpd36 at concentrations of 40 µM and 80 µM, and the lower chamber was filled with 10% FBS complete media. The cells were then incubated in a CO_2_ incubator for 48 h. For the invasion assay, we used Matrigel-coated membranes before cell seeding, and all other states remained the same as in the migration assay. After the incubation period, the Transwell was washed, and the non-migrated cells were removed using a cotton swab. The cells were fixed with 75% ethanol and stained with 0.5% crystal violet. Migrated and invaded cells were counted, and images were captured using a microscope.

### 4.9. Cell Cycle Analysis

5637, SCaBER, and MB49 cells were seeded at a density of 5 × 10^5^ cells/well in 6-well culture plates and incubated in a CO_2_ incubator. After 24 h, cells were exposed to different concentrations of Cpd36 (40 µM and 80 µM) for 48 h. After treatment, cells were collected and fixed with 70% chilled ethanol. The next day, ethanol was removed by centrifugation at 1500 rpm for 5 min, and the pellet was washed twice with PBS. The supernatant was removed, and the pellet was re-suspended in 500 µL FxCycle™ PI/RNase Staining Solution (Invitrogen™, Carlsbad, CA, USA # F10797) for 30 min and incubated in the dark at room temperature. The samples were processed using a FACS Caliber, and the data were analyzed using FlowJo software v11 using 532-nm excitation with a 585/42-nm bandpass filter.

### 4.10. Mitochondrial Membrane Potential

To measure the mitochondrial membrane potential, BCa cells 5637, SCaBER, and MB49 were counted and seeded at a density of 2 × 10^5^ cells per well in a 6-well culture plate. After attachment, the cells were treated with Cpd36 at 40 µM and 80 µM concentrations in the culture media for 48 h. After incubation, the cells were collected and stained with JC-1 dye (10 µL) according to the JC-1 Mitochondrial Membrane Potential Assay Kit (MedChemExpress NJ, USA # HY-K0601) and incubated in a CO_2_ incubator for 20 min. After incubation, the cells were centrifuged for 5 min at 1500 rpm and 4 °C, and the supernatant was carefully removed. The cell pellet was washed twice with PBS, suspended the cell pellet in 500 µL PBS, and analyzed using a flow cytometer. JC-1 monomers emit a green fluorescence peak at approximately 520 nm when excited at 490 nm. JC-1 aggregates exhibit orange-red fluorescence that peaks at approximately 590 nm.

### 4.11. Mitochondria ROS Production

MitoSOX™ Mitochondrial superoxide indicators (Invitrogen™ CA, USA # M36008) were employed to quantify the ROS production in BCa cells. 5637, SCaBER, and MB49 cells were seeded into a 6-well plate at 2 × 10^5^ cells per well. After 24 h, BCa cells were treated with different concentrations of Cpd36 for 48 h. Following incubation, cells were collected and stained with FACs buffer containing MitoSOX™ for 30 min in the dark. The sample was analyzed with flow cytometry. Excitation/emission wavelength 396 nm/610 nm.

### 4.12. Annexin V Staining

Apoptosis induction was determined using FITC Annexin V/Dead cell apoptosis kit (Invitrogen™ CA, USA # V13242). 5637, SCaBER, and MB49 cells were seeded at a density of 5 ×10^5^ cells/well in a six well plate and incubated in a CO_2_ incubator for 24 h. The next day, the cells were treated with Cpd36 at concentrations of 40 µM and 80 µM for 48 h. After completion of the treatment, the cells were harvested, washed with cold PBS, and resuspended in 1X annexin-binding buffer. First, 5 µL of FITC Annexin V and then 1 µL of PI (100 µg/mL) to each 100 µL of cell suspension were added and incubated at room temperature for 15 min. After the incubation period, 400 µL of 1X annexin-binding buffer was added and mixed carefully with a pipette, and the sample was analyzed by flow cytometry.

### 4.13. Western Blot Analysis

Cells were washed with cold PBS and subsequently added RIPA lysis and extraction buffer (Thermo Scientific™ CA, USA # 89900) containing both protease and phosphatase inhibitors for 30 min on ice. Then, cells were collected by scraping, and protein was quantified using the Pierce™ BCA protein assay kit (Thermo Scientific™ CA, USA # A55864). The protein concentrations of 10–30 µg per lane were resolved on a 10–15% SDS-PAGE and subsequently transferred onto a polyvinylidene difluoride (PVDF) membrane at 40 C. The membrane was blocked with 5% BSA at room temperature for 1–2 h and washed with 1X washing buffer and incubated with primary antibodies at 4 °C overnight and followed by incubation with secondary antibodies at room temperature for 1 h. The protein expression was detected using a Gbox instrument (Syngene, Frederick, MD, USA). The following primary and secondary antibodies were used to determine the expression of various proteins. The primary antibodies were used GALK1 (ABclonal-A15274, 1:1000, Woburn, MA, USA), PCNA (CST-13110, 1:1000, Danvers, MA, USA), Cleaved PARP (CST-5625, 1:1000), Cleaved Caspase 3 (CST-9664, 1:1000), AKT (CST-4691, 1:1000), pAkt (CST-4060, 1:1000), MMP-9 (ABclonal-A0289, 1:1000), p21(SC-397, 1:1000), p27(SC-528, 1:1000), Cyclin D1 (CST-55506, 1:1000), and rabbit secondary antibody (CST-7074, 1:10,000).

### 4.14. Statistical Analysis

Statistical analyses were performed using GraphPad Prism 10 software. Data was presented as mean ± SEM. Statistical significance was determined using *t*-tests with Welch’s correction. The significance levels are indicated as follows: * *p* < 0.05, ** *p* < 0.01, and *** *p* < 0.001.

## 5. Conclusions

In the present study, we demonstrated that GALK1 is upregulated in BCa and plays a crucial role in its development. Furthermore, we demonstrated that GALK1 inhibition with Cpd36 significantly inhibited cell proliferation, migration, invasion, cell cycle progression, and the induction of mitochondrial reactive oxygen species (mtROS) and apoptosis in BCa cells. In vitro, we found Cpd36 treatment altered expression of PCNA, Caspases, PARP, and AKT proteins that were associated with cell growth and cell death, further supporting the potential anticancer mechanism of GALK1 inhibition. Employing a synergistic approach with the chemotherapy drugs cisplatin and gemcitabine demonstrated that inhibiting GALK1 with Cpd36 increased the sensitivity to both drugs, presenting a new strategy for treating bladder cancer. Overall, our findings provide strong evidence that GALK1 inhibition is a promising therapeutic target for bladder cancer; therefore, further IND-enabling studies are needed to validate these findings in vivo.

## Data Availability

The original contributions presented in this study are included in this article. The raw data supporting the conclusions of this article will be made available by the authors on request.

## Abbreviations

The following abbreviations are used in this manuscript:

BCa	Bladder cancer
BCG	Bacillus Calmette-Guérin
DMSO	Dimethyl Sulfoxide
FBS	Fetal Bovine Serum
GALK1	Galactokinase-1
GEM	Gemcitabine
MIBC	Muscle-Invasive Bladder Cancer
mtROS	Mitochondrial Reactive Oxygen Species
mtMP	Mitochondrial membrane potential
OCT	Optical Coherence Tomography

## References

[1] Dyrskjøt L., Hansel D.E., Efstathiou J.A., Knowles M.A., Galsky M.D., Teoh J., Theodorescu D. (2023). Bladder Cancer. Nat. Rev. Dis. Primers.

[2] Siegel R.L., Kratzer T.B., Giaquinto A.N., Sung H., Jemal A. (2025). Cancer Statistics, 2025. CA A Cancer J. Clin..

[3] Yu Q., Li B., Lin H., Sun C., Yang X., Zhang Z. (2025). Smoking-Related Bladder Cancer Burden from 1990 to 2021: An Age-Period-Cohort Analysis of the Global Burden of Disease Study. Tob. Induc. Dis..

[4] Kumbham S., Rahman K.M.M., Foster B.A., You Y. (2025). A Comprehensive Review of Current Approaches in Bladder Cancer Treatment. ACS Pharmacol. Transl. Sci..

[5] Pectasides D., Pectasides M., Economopoulos T. (2006). Systemic Chemotherapy in Locally Advanced and/or Metastatic Bladder Cancer. Cancer Treat. Rev..

[6] Bellmunt J., von der Maase H., Mead G.M., Skoneczna I., De Santis M., Daugaard G., Boehle A., Chevreau C., Paz-Ares L., Laufman L.R. (2012). Randomized Phase III Study Comparing Paclitaxel/Cisplatin/Gemcitabine and Gemcitabine/Cisplatin in Patients with Locally Advanced or Metastatic Urothelial Cancer without Prior Systemic Therapy: EORTC Intergroup Study 30987. J. Clin. Oncol. Off. J. Am. Soc. Clin. Oncol..

[7] Martini A., Lonati C., Montorsi F., Briganti A., Colombo R., Necchi A., Simeone C., Zamboni S., Afferi L., Mattei A. (2021). The Role of Prior Bladder Cancer on Recurrence in Patients Treated with Radical Nephroureterectomy. Clin. Genitourin. Cancer.

[8] Jang M., Kim S.S., Lee J. (2013). Cancer Cell Metabolism: Implications for Therapeutic Targets. Exp. Mol. Med..

[9] Ward P.S., Thompson C.B. (2012). Metabolic Reprogramming: A Cancer Hallmark Even Warburg Did Not Anticipate. Cancer Cell.

[10] Woolbright B.L., Ayres M., Taylor J.A. (2018). Metabolic Changes in Bladder Cancer. Urol. Oncol. Semin. Orig. Investig..

[11] Conte F., van Buuringen N., Voermans N.C., Lefeber D.J. (2021). Galactose in Human Metabolism, Glycosylation and Congenital Metabolic Diseases: Time for a Closer Look. Biochim. Biophys. Acta (BBA)—Gen. Subj..

[12] Barretina J., Caponigro G., Stransky N., Venkatesan K., Margolin A.A., Kim S., Wilson C.J., Lehár J., Kryukov G.V., Sonkin D. (2012). The Cancer Cell Line Encyclopedia Enables Predictive Modelling of Anticancer Drug Sensitivity. Nature.

[13] Sharpe M.A., Ijare O.B., Raghavan S., Baskin A.M., Baskin B.N., Baskin D.S. (2024). Targeting the Leloir Pathway with Galactose-Based Antimetabolites in Glioblastoma. Cancers.

[14] Davalieva K., Kiprijanovska S., Ivanovski O., Trifunovsk A., Saidi S., Dimovski A., Popov Z. (2023). Proteomics Profiling of Bladder Cancer Tissues from Early to Advanced Stages Reveals NNMT and GALK1 as Biomarkers for Early Detection and Prognosis of BCa. Int. J. Mol. Sci..

[15] Liu L., Tang M., Pragani R., Whitby F.G., Zhang Y., Balakrishnan B., Fang Y., Karavadhi S., Tao D., LeClair C.A. (2021). Structure-Based Optimization of Small Molecule Human Galactokinase Inhibitors. J. Med. Chem..

[16] Tang M., Etokidem E., Lai K. (2016). The Leloir Pathway of Galactose Metabolism—A Novel Therapeutic Target for Hepatocellular Carcinoma. Anticancer Res..

[17] Huang R., Li M., Zeng Z., Zhang J., Song D., Hu P., Yan P., Xian S., Zhu X., Chang Z. (2022). The Identification of Prognostic and Metastatic Alternative Splicing in Skin Cutaneous Melanoma. Cancer Control..

[18] Han X., Han B., Luo H., Ling H., Hu X. (2023). Integrated Multi-Omics Profiling of Young Breast Cancer Patients Reveals a Correlation between Galactose Metabolism Pathway and Poor Disease-Free Survival. Cancers.

[19] Wang T., Wang Z. (2025). Targeting the “Undruggable”: Small-Molecule Inhibitors of Proliferating Cell Nuclear Antigen (PCNA) in the Spotlight in Cancer Therapy. J. Med. Chem..

[20] Augoff K., Hryniewicz-Jankowska A., Tabola R., Stach K. (2022). MMP9: A Tough Target for Targeted Therapy for Cancer. Cancers.

[21] Reis S.T., Leite K.R.M., Piovesan L.F., Pontes-Junior J., Viana N.I., Abe D.K., Crippa A., Moura C.M., Adonias S.P., Srougi M. (2012). Increased Expression of MMP-9 and IL-8 Are Correlated with Poor Prognosis of Bladder Cancer. BMC Urol..

[22] Fares J., Fares M.Y., Khachfe H.H., Salhab H.A., Fares Y. (2020). Molecular Principles of Metastasis: A Hallmark of Cancer Revisited. Signal Transduct. Target. Ther..

[23] Singh S., Pathuri G., Asch A., Rao C. (2024). Venkateshwar Madka Stat3 Inhibitors TTI-101 and SH5-07 Suppress Bladder Cancer Cell Survival in 3D Tumor Models. Cells.

[24] Folkesson E., Niederdorfer B., Nakstad V.T., Thommesen L., Klinkenberg G., Lægreid A., Flobak Å. (2020). High-Throughput Screening Reveals Higher Synergistic Effect of MEK Inhibitor Combinations in Colon Cancer Spheroids. Sci. Rep..

[25] Lagies S., Schlimpert M., Neumann S., Wäldin A., Kammerer B., Borner C., Peintner L. (2020). Cells Grown in Three-Dimensional Spheroids Mirror in Vivo Metabolic Response of Epithelial Cells. Commun. Biol..

[26] Pucci B., Kasten M., Giordano A. (2000). Cell Cycle and Apoptosis. Neoplasia.

[27] Fang Y., Cao Z., Hou Q., Ma C., Yao C., Li J., Wu X.-R., Huang C. (2013). Cyclin D1 Downregulation Contributes to Anticancer Effect of Isorhapontigenin on Human Bladder Cancer Cells. Mol. Cancer Ther..

[28] Sarsik B., Doganavsargil B., Simsir A., Yazici A., Pehlivanoglu B., Cal C., Sen S. (2016). P21 and P27 Immunoexpression in Upper Urinary Tract Urothelial Carcinomas. Pathol. Oncol. Res. POR.

[29] Roy S., Gu M., Ramasamy K., Singh R.P., Agarwal C., Siriwardana S., Sclafani R.A., Agarwal R. (2009). P21/Cip1 and P27/Kip1 Are Essential Molecular Targets of Inositol Hexaphosphate for Its Antitumor Efficacy against Prostate Cancer. Cancer Res..

[30] Lin W., Li Y., Huang H., Zhao P., Su Y., Fang C.-Y. (2025). Harmine Hydrochloride Induces G0/G1 Cell Cycle Arrest and Apoptosis in Oral Squamous Carcinoma Cells. Exp. Ther. Med..

[31] Almalki S.G. (2023). The Pathophysiology of the Cell Cycle in Cancer and Treatment Strategies Using Various Cell Cycle Checkpoint Inhibitors. Pathol.—Res. Pract..

[32] Peng B.-Y., Singh A.K., Chan C.-H., Deng Y.-H., Li P.-Y., Su C.-W., Wu C.-Y., Deng W.-P. (2023). AGA Induces Sub-G1 Cell Cycle Arrest and Apoptosis in Human Colon Cancer Cells through P53-Independent/P53-Dependent Pathway. BMC Cancer.

[33] Dho S.H., Cho M., Woo W., Jeong S., Kim L.K. (2025). Caspases as Master Regulators of Programmed Cell Death: Apoptosis, Pyroptosis and Beyond. Exp. Mol. Med..

[34] Suski J.M., Lebiedzinska M., Bonora M., Pinton P., Duszynski J., Wieckowski M.R. (2012). Relation between Mitochondrial Membrane Potential and ROS Formation. Methods Mol. Biol..

[35] Revathidevi S., Munirajan A.K. (2019). Akt in Cancer: Mediator and More. Semin. Cancer Biol..

[36] Hassan D., Menges C.W., Testa J.R., Bellacosa A. (2024). AKT Kinases as Therapeutic Targets. J. Exp. Clin. Cancer Res..

[37] Szanto A., Bognar Z., Szigeti A., Szabo A., Farkas L., Gallyas F. (2009). Critical Role of Bad Phosphorylation by Akt in Cytostatic Resistance of Human Bladder Cancer Cells. Anticancer Res..

[38] Tan Y., Li J., Zhao G., Huang K.-C., Cardenas H., Wang Y., Matei D., Cheng J.-X. (2022). Metabolic Reprogramming from Glycolysis to Fatty Acid Uptake and Beta-Oxidation in Platinum-Resistant Cancer Cells. Nat. Commun..

[39] Wen H., Lee S., Zhu W.-G., Lee O.-J., Yun S.J., Kim J., Park S. (2019). Glucose-Derived Acetate and ACSS2 as Key Players in Cisplatin Resistance in Bladder Cancer. Biochim. Biophys. Acta Mol. Cell Biol. Lipids.

[40] Kaufman D.S., Shipley W.U., Feldman A.S. (2009). Bladder Cancer. Lancet.

[41] Sternberg C.N., Bellmunt J., Sonpavde G., Siefker-Radtke A.O., Stadler W.M., Bajorin D.F., Dreicer R., George D.J., Milowsky M.I., Theodorescu D. (2012). ICUD-EAU International Consultation on Bladder Cancer 2012: Chemotherapy for Urothelial Carcinoma—Neoadjuvant and Adjuvant Settings. Eur. Urol..

[42] Chandrashekar D.S., Karthikeyan S.K., Korla P.K., Patel H., Shovon A.R., Athar M., Netto G.J., Qin Z.S., Kumar S., Manne U. (2022). UALCAN: An update to the integrated cancer data analysis platform. Neoplasia.

[43] Yan F., Ha J.-H., Yan Y., Ton S.B., Wang C., Mutembei B., Alhajeri Z.A., McNiel A.F., Keddissi A.J., Zhang Q. (2022). Optical Coherence Tomography of Tumor Spheroids Identifies Candidates for Drug Repurposing in Ovarian Cancer. IEEE Trans. Biomed. Eng..

